# Phenotypic intrafamilial variability including H syndrome and Rosai–Dorfman disease associated with the same c.1088G > A mutation in the *SLC29A3* gene

**DOI:** 10.1186/s40246-021-00362-z

**Published:** 2021-10-17

**Authors:** Hamza Chouk, Mohamed Ben Rejeb, Lobna Boussofara, Haїfa Elmabrouk, Najet Ghariani, Badreddine Sriha, Ali Saad, Dorra H’Mida, Mohamed Denguezli

**Affiliations:** 1 Higher Institute of Biotechnology of Monastir, University of Monastir, Monastir, Tunisia; 2 Human Cytogenetics, Molecular Genetics and Reproductive Biology, Department of Genetics, Farhat Hached University Hospital of Sousse, Sousse, Tunisia; 3 Department of Dermatology, Farhat Hached University of Sousse, Sousse, Tunisia; 4 Faculty of Medicine of Sousse, University of Sousse, Sousse, Tunisia; 5 Department of Pathological Anatomy, Farhat Hached University Hospital of Sousse, Sousse, Tunisia

**Keywords:** *SLC29A3* gene, H syndrome, Rosai–Dorfman disease, Histiocytosis, Genodermatosis, hENT3, Intrafamilial variability

## Abstract

**Background:**

Mutations in the *SLC29A3* gene, which encodes the nucleoside transporter hENT3, have been implicated in syndromic forms of histiocytosis including H syndrome, pigmented hypertrichosis with insulin-dependent diabetes, Faisalabad histiocytosis and Familial Rosai–Dorfman disease (RDD). Herein, we report five new patients from a single family who present with phenotypes that associate features of H syndrome and Familial Rosai–Dorfman disease.

**Methods:**

We investigated the clinical, biochemical, histopathological and molecular findings in five Tunisian family members' diagnosed with Familial RDD and/or H syndrome. The solute carrier family 29 (nucleoside transporters), member 3 (*SLC29A3*) gene was screened for molecular diagnosis using direct Sanger sequencing.

**Results:**

Genetic analysis of all affected individuals revealed a previously reported missense mutation c.1088 G > A [p.Arg363Gln] in exon 6 of the *SLC29A3* gene. Four affected members presented with clinical features consistent with the classical H syndrome phenotype. While their cousin’s features were in keeping with Familial Rosai–Dorfman disease diagnosis with a previously undescribed cutaneous RDD presenting as erythematous nodular plaques on the face. This report underlines the clinical variability of *SLC29A3* disorders even with an identical mutation in the same family.

**Conclusion:**

We report a rare event of 5 Tunisian family members' found to be homozygous for *SLC29A3* gene mutations but showing a different phenotype severity. Our study reveals that despite a single mutation, the clinical expression of the *SLC29A3* disorders may be significantly heterogeneous suggesting a poor genotype–phenotype correlation for the disease.

## Introduction

The histiocytoses are a group of systemic disorders characterized by an excessive number of histiocytes [1]. The etiology and pathogenesis of histiocytic disorders remain broadly unclear [1, 2]. The *SLC29A3* gene has recently been found mutated in rare patients with inherited syndromic histiocytosis resulting in 4 overlapping phenotypes including H syndrome, pigmented hypertrichosis with insulin-dependent diabetes mellitus (PHID), Faisalabad histiocytosis (FHC) and sinus histiocytosis with massive lymphadenopathy (SHML) or Familial Rosai–Dorfman disease (RDD) [3]. The present study highlights a remarkable intrafamilial phenotypic variability of *SLC29A3* disorder among 5 members of a Tunisian family harboring the same mutation in the *SLC29A3* gene and presenting with overlapping features of H syndrome and Familial RDD.

## Methods

After obtaining informed consents, we collected the clinical, biochemical, histopathological, and genetic characteristics of 5 affected members of a Tunisian family presenting with a clinical spectrum of *SLC29A3* gene defects. Genomic DNA was isolated from peripheral blood leucocytes by standard techniques. Mutation analysis was carried out in all six exons and exon–intron boundaries of the *SLC29A3* gene by direct sequencing.

## Results

### Clinical investigation

**Fig. 1:**
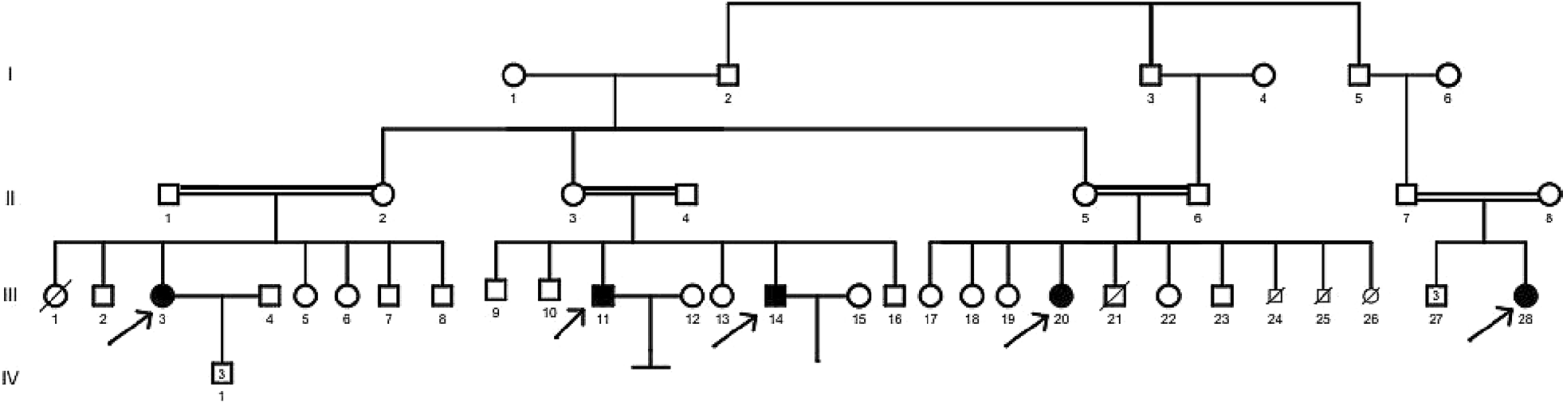
Pedigree of the family with phenotypic heterogeneity associating H syndrome and RDD: H syndrome patients (III.3, III.11, III.14, and III.28), RDD patient (III.20)

This study reports a family from Kairouan rural area in central, Tunisia. As shown in Fig. 1, five affected individuals are included in the same consanguineous family (2 brothers and 3 first cousins) with a common ancestor. Their ages ranged between 46 and 63 years. We have identified 4 patients with H syndrome and 1 patient with Familial RDD. The clinical data and laboratory investigations of the patients are summarized in Table 1.

**Table 1:** Summary of clinical, histological and genetics findings in our Tunisian family

Clinical features	Patient III.3	Patient III.11	Patient III.14	Patient III.28	Patient III.20
Gender	F	M	M	F	F
Age at diagnosis	63	54	46	52	51
Cutaneous hyperpigmentation	+	+	+	+	+
Hypertrichosis/sclerosis	+/+	+/+	+/+	+/+	−/−
Erythematous nodules	−	−	−	−	+
Location	Thighs legs	Lower back	Thighs/legs abdomen	Thighs legs	Face
Flexions contractures/Hallux valgus	+/+	+/+	+/+	+/+	−
Hearing loss	−	+	+	−	+
Insulin-dependent diabetes mellitus	−	−	+	−	−
Short stature	−	−	−	−	−
HSMG	−	−	−	−	−
Dilated scleral vessels	−	−	+	−	−
Lymphadenopathy	−	−	−	−	+
Echocardiogram	Cardiomegaly pericardial effusion	−	−	−	−
Anemia	+	−	−	+	+
Histopathological findings	Striking infiltrates of CD68+, CD1a−, PS100-histiocytes and cd138 + plasma cells				Inflammatory cell infiltrate, rich in plasma cells and histiocytes (CD68+, PS100+, CD1a−) which showed emperipolesis
*SLC29A3* Mutation	c.1088G > A: p.Arg363Gln				
SHL + Phenotype	**H syndrome**				**Familial RDD**

F: Female, M: male, −: absent, + : present,

### Patients: III.3, III.11, III.14, III.28

**Fig. 2:**
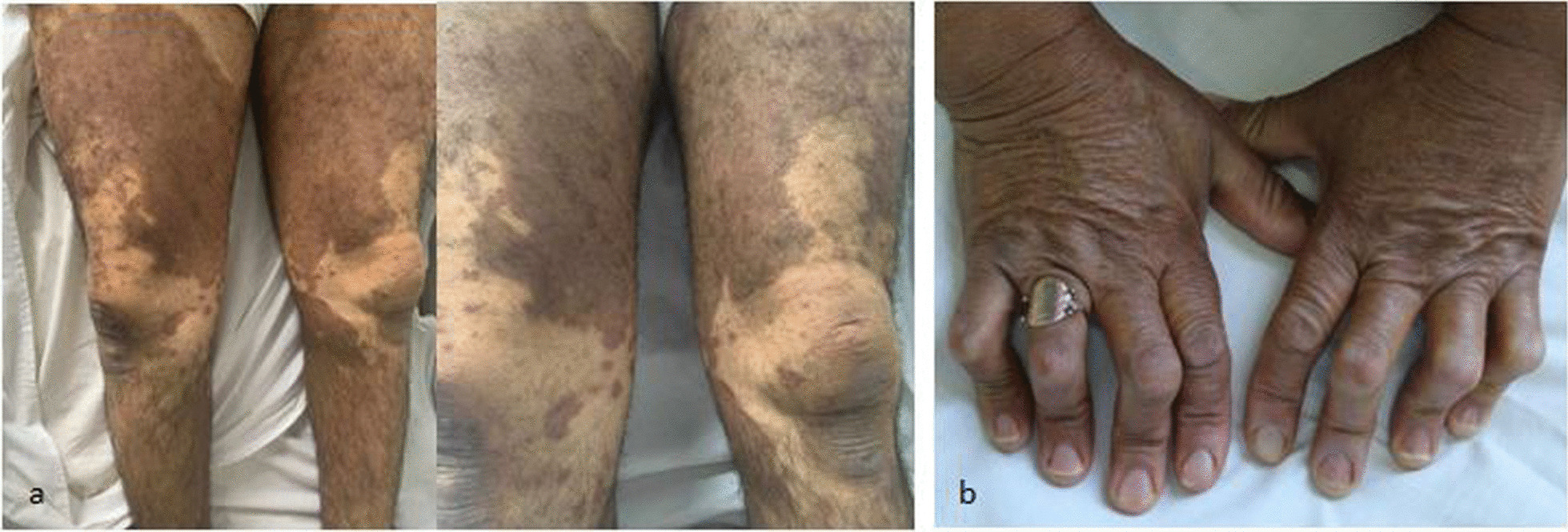
H syndrome. **a** Large, hyperpigmented, and hypertrichotic skin patches involving symmetrically the inner aspects of both thighs and shins. **b** Multidigit camptodactyly of the hands

Two females and two males patients from a consanguineous marriage were identified with a median age at diagnosis of 53 years. The two brothers (III.11, III.14) experienced a bilateral hearing loss that started during the pediatric period. The most common clinical finding is cutaneous hyperpigmentation, accompanied by hypertrichosis and induration observed in all patients. The average age of onset of skin lesions is 15 years. The lower limbs were affected in 3 cases (III.3, III.14, III.28), mainly in the thighs’ region and legs (Fig. 2a). Patient III.11 had only pigmented skin patches on the lower back. There were additional hyperpigmented patches over the abdomen in one case (III.14). Other cutaneous features including xerosis and keratosis pilaris were observed in patient III.28. Skeletal examination revealed fixed flexion contractures of the second to fifth PIP joints of hands (Fig. 2b) and feet with hallux valgus in feet in all cases. There was no hepatosplenomegaly or lymphadenopathy. No gynecomastia or genital swellings were detected. Ophthalmic examination of *patient* III.14 revealed dilated lateral scleral vessels. He was also diagnosed with insulin-dependent diabetes mellitus*.* Audiometric evaluation of hearing impairment confirmed bilateral perceptive deafness in male patients (III.11, III.14). Echocardiogram of patient III.3 revealed cardiomegaly with moderate pericardial effusion. Laboratory investigations showed microcytic anemia in 2 cases (III.3, III28), and inflammatory markers were elevated including erythrocyte sedimentation rate (100 mm/h) and C-reactive protein (70 mg/dL) in one case (III.28). Antinuclear and anti-double-stranded DNA antibodies were negative in all patients.

**Fig. 3:**
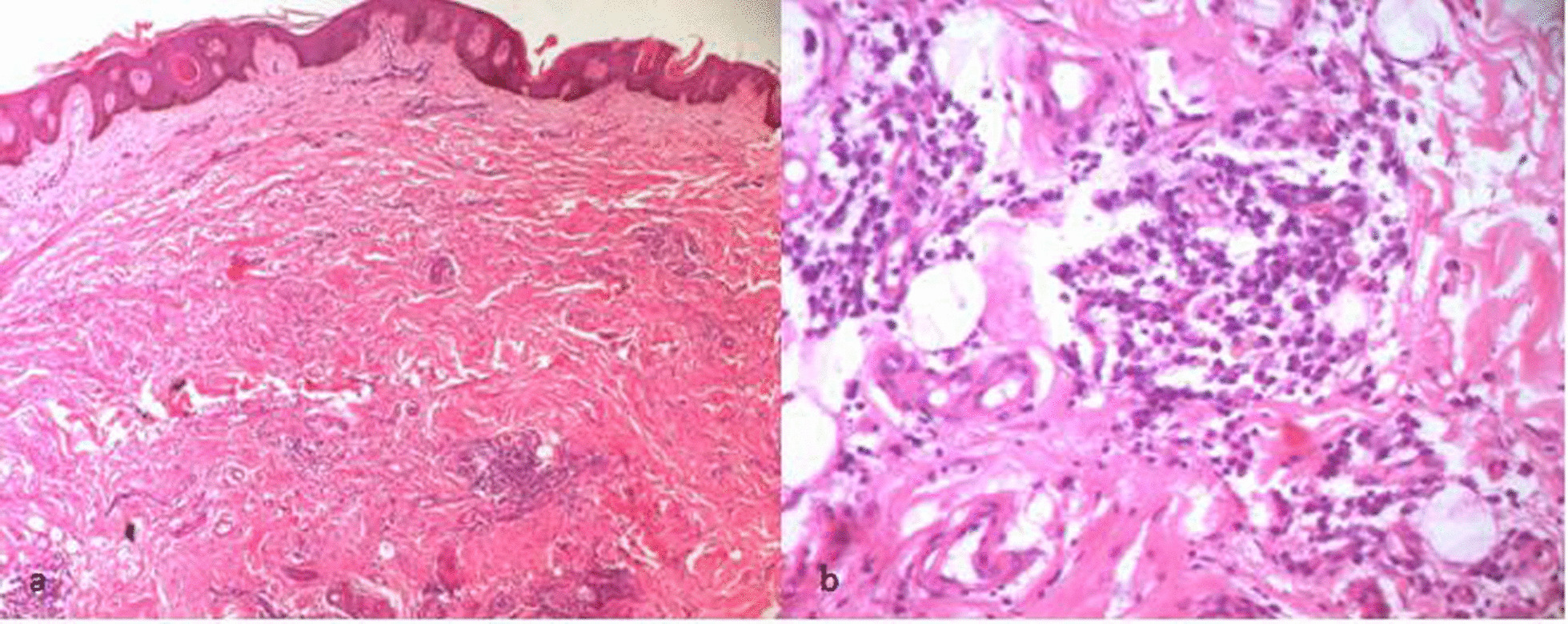
H syndrome. The dermis contains a spread fibrosis with an inflammatory perivascular infiltrate (**a**, HE*40) composed of mainly plasma cells, small histiocytes and lymphocytes (**b**, HE*200). Basal layer hyperpigmentation in the epidermis was also noted (**a**, HE*40)

**Fig. 4:**
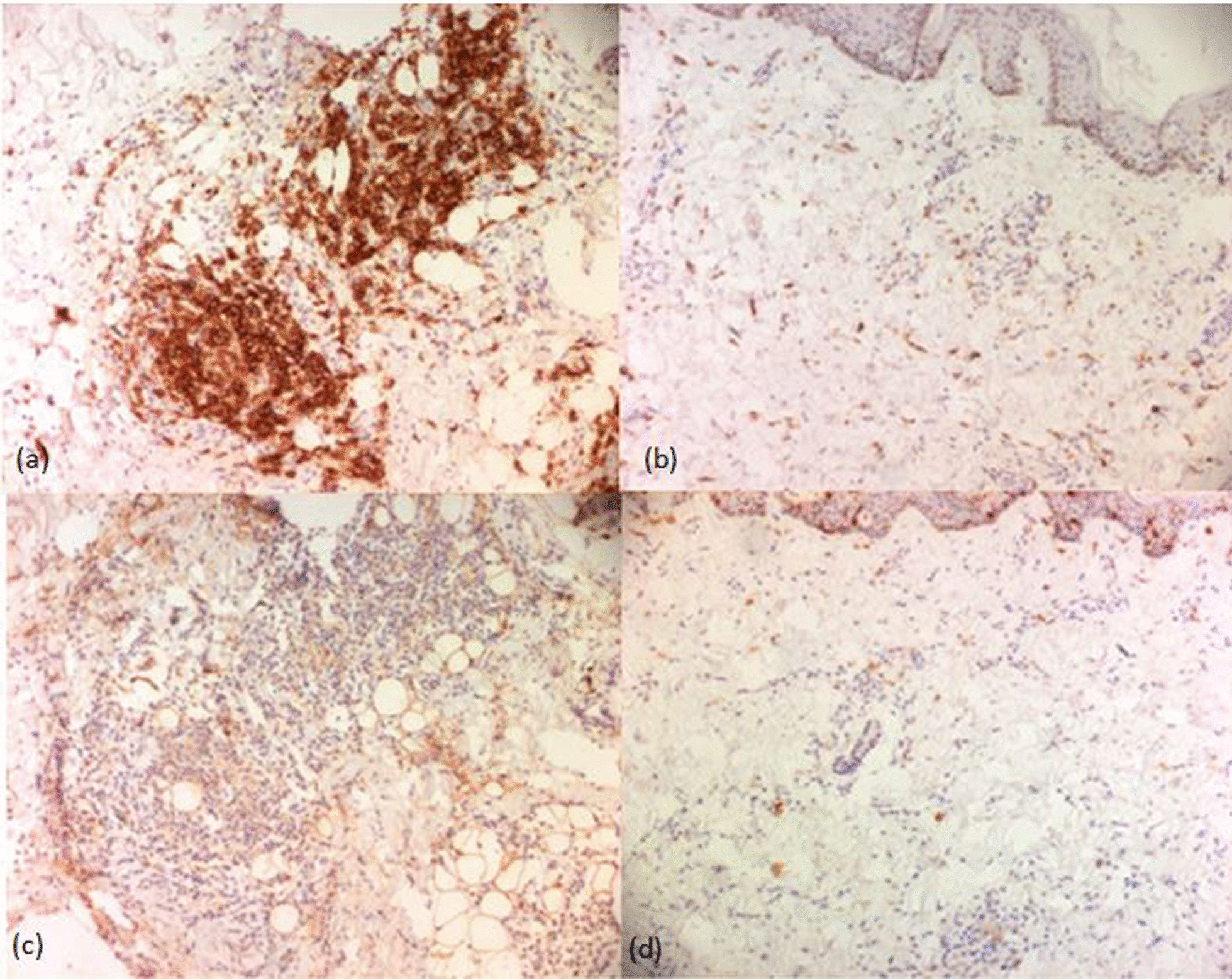
H syndrome. Immunohistochemistry stain shows diffuse infiltration plasma cells (**a**: CD 138+, × 100) with a moderate infiltration of histiocytes (**b**: CD68+, × 100). These histiocytes were not highlighted by immunohistochemical staining for S-100 protein (**c**) and CD1a (**d**) (× 100)

**Fig. 5:**
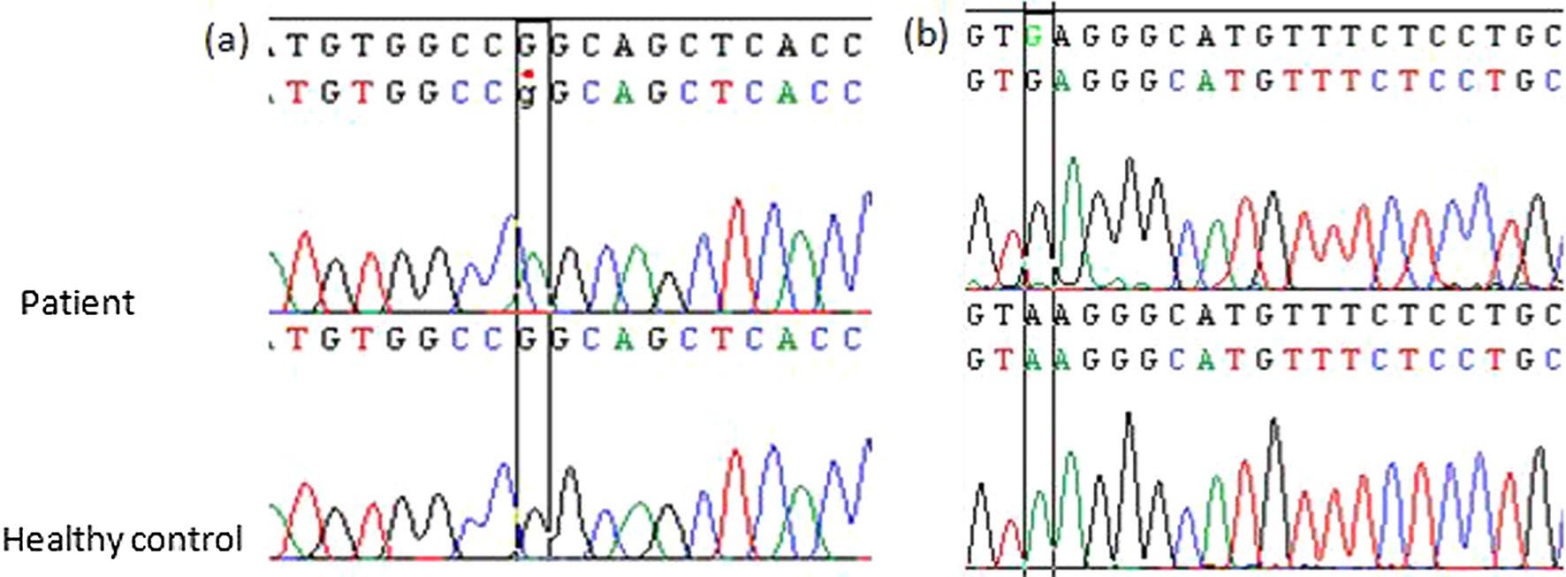
Sanger sequencing profile of our patients showing: **a** homozygous substitution c.1088G > A [p.Arg363Gln] mutation compared to a healthy control; **b** non-pathogenic variant c.300 + 3A > G at the homozygous state compared to a healthy control

Histopathological examination of a skin biopsy from a hyperpigmented area revealed the same characteristics in all affected family members. It was indeed an abundant melanin deposition in basal keratinocytes with spread fibrosis throughout the dermis and hypodermis. In the dermis, we found a prominent inflammatory perivascular infiltrate composed of plasma cells, small histiocytes and lymphocytes with a clear predominance of plasma cells (Fig. 3). There were no foamy histiocytes or emperipolesis. Immunohistochemical staining of the specimen showed interstitial CD68 + histiocytic (Fig. 4b). These histiocytes were not highlighted by immunohistochemical staining for S-100 protein and CD1a (Fig. 4c, d). Immunohistochemical staining for plasma cells markers showed diffuse staining of cells for CD138 (Fig. 4a). *SLC29A3* gene mutation analysis of family members showed a previously reported missense mutation c.1088 G > A [p.Arg363Gln] in exon 6 (Fig. 5a). We identified a benign splice site variant c.300 + 3A > G located in intron 2 in the *SLC29A3* gene (Fig. 5b).

### Patient: III.20

**Fig. 6:**
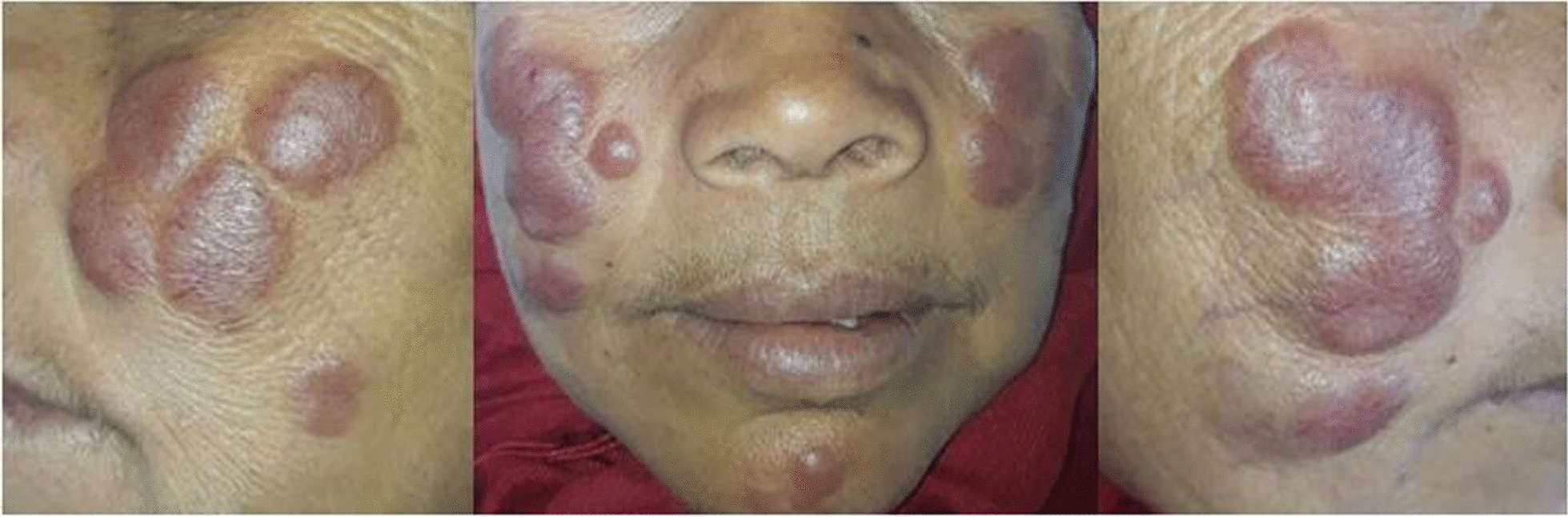
Rosai–Dorfman disease. Patient III.20 with bilateral erythematous to purplish, well-defined, nodules on her face

**Fig. 7:**
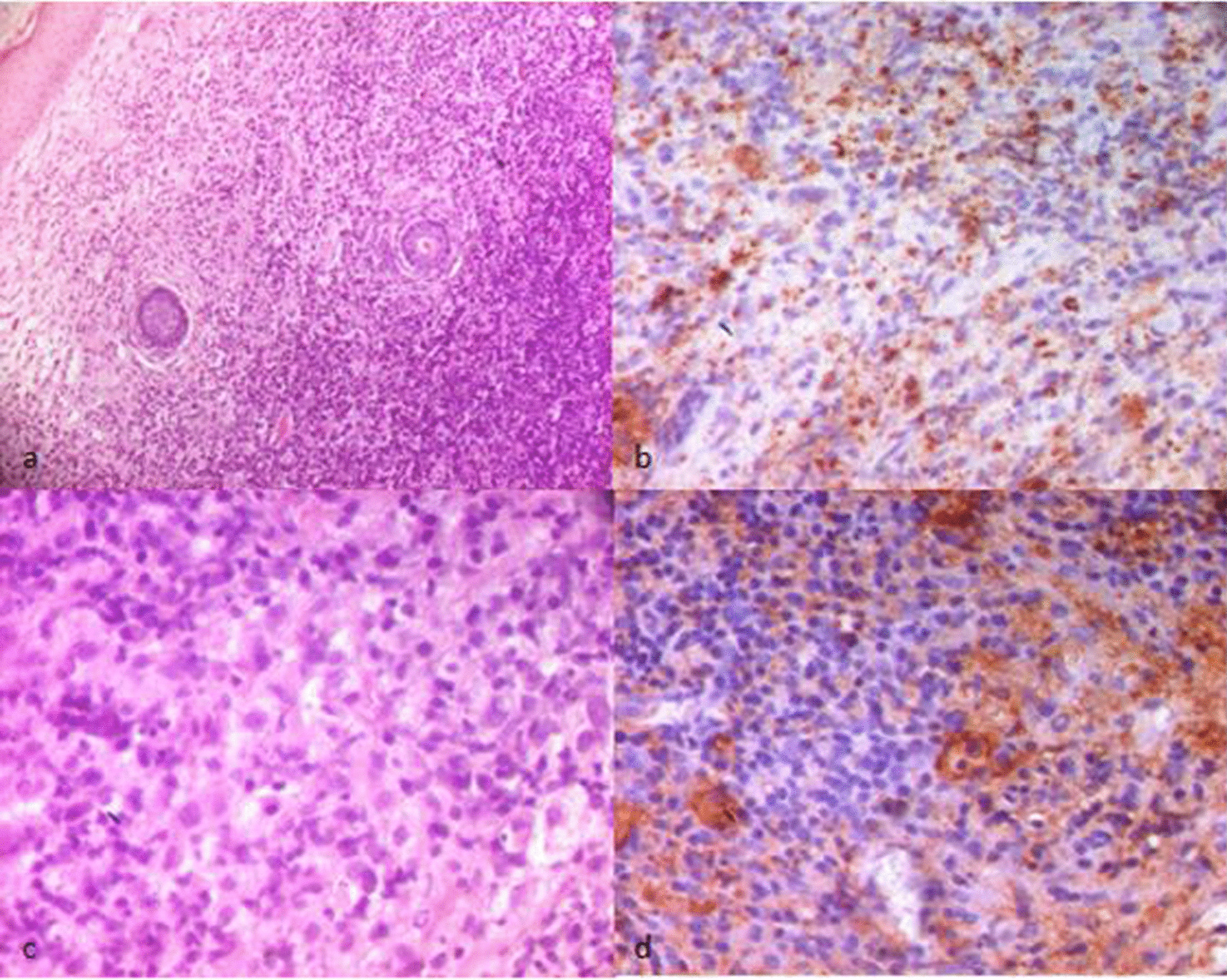
Rosai–Dorfman disease. Histopathological findings of the skin biopsy from the patient III.20 showed a dense inflammatory cell infiltrate, rich in plasma cells and histiocytes (**a**, HE*40), stained positive with CD68 (**b**, × 400) and S-100 protein (**d**, × 400) immunohistochemistry with emperipolesis/lymphocytophagocytosis (**c**, HE*400)

A 51-year-old woman was born to consanguineous healthy parents. Four members of her family described above were diagnosed with H syndrome. Her five siblings were healthy, without any skin abnormalities. She presented with a 10-year history of multiple nodules on her face. First lesions appeared as small asymptomatic red, nodules on her chin and forehead, the number and size of which slowly increased over time. Her medical history was otherwise significant for unexplored hearing loss and anemia since 15 year old. She recently reported progressive dysphonia and nasal obstruction. However, there was no history of diabetes mellitus, nor delayed puberty. Dermatological examination revealed indurated bilateral, erythematous to purplish, well-defined, nodules on her face particularly over her cheeks measuring 1 to 6 cm in diameter (Fig. 6). In contrast to her cousins, there were no associated pigmented skin lesions. Physical examination revealed bilateral enlargement of axillary and inguinal lymph nodes, all of which were firm and painless on palpation. There was no fever, nor osteoarticular deformations, nor organomegaly with normal stature. Ophthalmologic and neurological evaluations were normal. Small budding lesions filling the nasal cavities were detected via an anterior rhinoscopic examination. An audiogram concluded a bilateral sensorineural hearing loss. Laboratory tests revealed hemoglobin of 7 g/dL, with a normal total leukocyte count and platelets. Peripheral smear was consistent with microcytic, hypochromic anemia, related to iron deficiency. ESR (80 mm/h) and CRP (70 mg/dL) were high. Serum protein electrophoresis showed polyclonal hypergammaglobulinemia. Blood glucose, renal and liver functions were normal. The histopathology of the skin lesion exhibited a dense mixed inflammatory cell infiltrate, rich in plasma cells and histiocytes, which showed emperipolesis (Fig. 7a). Immunohistochemical studies revealed histiocytes with positivity for S100 and CD68 and negativity for CD1a (Fig. 7). Therefore, the diagnosis of extranodal RDD was made with a cutaneous, nasal and lymph nodes involvement. Sequence analysis revealed the same homozygous missense mutation c.1088 G > A [p.Arg363Gln] in the exon 6 as here H syndrome family members.

## Discussion

The *SLC29A3* gene encodes the human equilibrative nucleoside transporter, hENT3, a mitochondrial and a lysosomal nucleoside transporter which mediates intracellular salvage of hydrophilic nucleosides [4]. The loss of hENT3 has been associated with clinical spectrum disorders of *SLC29A3* gene defects, also known as histiocytosis-lymphadenopathy plus syndrome comprising features of 4 histiocytic disorders sharing overlapping clinical manifestations previously thought to be distinct: Faisalabad histiocytosis (FHC), sinus histiocytosis with massive lymphadenopathy (SHML) or Familial Rosai–Dorfman disease (RDD), H syndrome, and pigmented hypertrichosis with insulin-dependent diabetes mellitus syndrome (PHID) [3, 5, 6]. The first condition to be associated with recessive mutations in the *SLC29A3* gene was the H syndrome [7]. To our knowledge, almost 100 patients worldwide have been confirmed to have H syndrome [8]. Patients with this syndrome were mostly described in families of Arab origin from consanguineous parents [8, 9]. H syndrome clinical presentation is variable in its spectrum and degree of severity. It is characterized by major clinical findings of cutaneous hyperpigmentation, with hypertrichosis and concomitant diverse systemic manifestations that include hearing loss, flexion contractures/hallux valgus, short stature, hypogonadism, hepatosplenomegaly, cardiomyopathy, and potential hyperglycemia [8, 10]. The common clinical manifestations of H syndrome, and the hallmarks for the diagnosis, are large hyperpigmented and hypertrichotic skin lesions, which are symmetrically involving the lower part of the body typically in the medial areas of the thighs, camptodactyly, sensorineural hearing loss and short stature [11, 12]. As in our case, cutaneous hyperpigmentation was the hallmark of H syndrome, and it was described in all four patients. Other features such as genital masses, hallux valgus, hepatomegaly and splenomegaly, gynecomastia, pericardial effusion, exophthalmos, dilated lateral scleral vessels and facial telangiectasia, are present in less than 30% of patients [13]. Recently, several novel features have been recognized including premature graying of the hair, bilateral optic disc swelling, hypospadias, retroperitoneal fibrosis, cardiogenic shock, digital ischemia, Raynaud's phenomenon and generalized lipoatrophy [10, 14–16, 26]. As shown in Table 1, our patients exhibit several manifestations including cutaneous pigmentation, flexion contractures, and hearing loss. Although short stature was reported in mostly H syndrome reports, our four patients have a normal height [8]. H syndrome clinical presentation can also vary within the same family [13], as illustrated by the intrafamilial phenotypic variability in our 4 related patients.

Standardized histopathological skin lesions criteria proposed by Doviner et al., consist of the presence of hyperpigmentation of basal layer; widespread fibrosis of dermis and mononuclear cell infiltrates consisting mainly of monocyte-derived cells (small CD68 + histiocytes and CD34 + FXIIIa + dendrocytes), and plasma cells [17]. H syndrome histological findings in our cases showed an inflammatory dermal infiltrate rich in plasma cells (CD138 +). This atypical histological presentation is increasingly described in the literature [10].

Based on the above clinical and histological descriptions, the diagnosis of H syndrome should be strongly suspected and confirmed by the molecular study. In contrast with H syndrome, the Familial RDD phenotype was only twice associated with mutations in the *SLC29A3* gene [3]. The first case was identified by Morgan et al. [3] Turkish brothers previously studied by Kismet et al. with features of cervical, lymphadenopathy, short stature, hepatomegaly sensorineural deafness and orbital mass [3, 18]. Jonard et al. reported in a 17-year-old Moroccan girl with overlapping features of Familial RDD and H syndrome presenting with only a progressive sensorineural hearing impairment and a single cervical node [19]. Herein, we report an additional Familial RDD case in 54-year-old Tunisian women with a prominent cutaneous feature not previously described. Our patient is the first case of Familial RDD with clinical features of cutaneous RDD including erythematous nodular plaques on the face. Interestingly, cutaneous lesions, found in our patient, are similar to those described in the sporadic form of RDD. Deafness and lymphadenopathy were observed in our patient which is in line with those reported by Morgan et al. [3] and Jonard et al. [19]. Following our observation, cutaneous pigmentation and flexion contractures have not been reported in Familial RDD patients. However, sensorineural hearing loss is present in both Familial RDD and H syndrome [3, 19].

Histologically, the hallmark of Familial RDD is emperipolesis which is generally absent in H syndrome [3]. As seen in our cases, mutations in *SLC29A3* have been found to cause a wide variety of phenotypes. To our knowledge, more than 22 pathogenic or likely pathogenic variants have been identified in the *SLC29A3* gene [4] (Table 2). The 2 most common mutations reported in H syndrome are c.1309G > A; [p.Gly437Arg] and c.1279G > A; [p.Gly427Ser] [8]. Nine out of 25 reported mutations were found in the 6th exon of the gene highlighting the effectiveness of initial screening of this coding region for mutation’s detection in *SLC29A3* [5, 13].

**Table 2:** H Syndrome *SLC29A3* reported mutations

Nucleotide change	Localization	Amino acid change	Family origin	Reference
c.1088G > A	Exon 6	p.Arg363Gln	Tunisia	Melki et al.
			Tunisia	Jaouadi et al.
			Spanish/Morocco	Molho et al.
c.1087C > T	Exon 6	p.Arg363Trp	Mexico	Bloom et al.
			ND	Molho et al.
c.401G > A	Exon 4	p.Arg134His	Egypt	Al-Haggar et al.
c.610 + 1G > A	Intron 4	p ^NA^	ND	Bloom et al.
c.307-308delTT	Exon 3	p.Phe103fs	Iran	Noavar et al.
c.625G > A	Exon 5	p.Gly208Arg	Japan	Fujita et al.
c.1309G > A	Exon 6	p.Gly437Arg	Israel	Molho et al.
			India	Priya et al.
			ND	Ramot et al.
			Israel	Spiegel et al.
			Egypt	El-Bassyouni
c.552C > G	Exon 4	p.Ser184Arg	Japan	Ramot et al.
IVS1 + 2T > G	Intron 1	p ^NA^	Syria	Farooq et al.
c.971C > T	Exon6	p.Pro324Leu	Tunisia	Jaouadi et al.
			Morocco	Molho et al.
c.42delC	Exon2	p.S15Pfs*86	Tunisia	Jaouadi et al.
c.243delA/c.300 + 1G > C	Exon 2	p.K81Nfs/p.N101LfsX34	Morocco	Bakhchane et al.
c.1279G > A	Exon 6	p.Gly427Ser	Bulgaria	Molho et al.
			Egypt	Al-Haggar et al.
			Israel	Spiegel et al.
c.400C > T	Exon 4	p.Arg134Cys	India	Mruthyunjaya et al.
c.1045delC	Exon 6	p.Leu349Serfs	ND	Molho et al.
c.933T > A	Exon 6	p.C310*	Turkey	Mutlu et al.
c.347T > G/c.610 + 1G > C	Exon3/Intron 4	p.Met116Arg/p ^NA^	ND	Bloom et al.
c.1269_1270delinsA	Exon 6	p.Leu424Serfs*29	China	Liu et al.
c.1339G > A	Exon 6	p.Glu447Lys	Turkey	Vural et al.
c.300 + 2T > G	Intron 2	p ^NA^	Syria	Farooq et al.
c.300 + 1G > A	Intron 2	p ^NA^	ND	Morgan et al.
c.155G > A/c.1309G > A	Exon3/Exon6	p ^NA^/p.Gly437Arg	Iran	Darvish et al.

p.^NA^: Non-applicable protein, ND: not determined

We report a missense homozygous mutation in exon 6 c.1088G > A, [p.Arg363Gln] changing Arginine to Glutamine which is found in a highly evolutionarily conserved position in the protein between the 8th and the 9th transmembrane domains. This same mutation was for the first time reported by Molho Pessach et al. in a 13-year-old Spanish boy and a 20-year-old Arab man with H syndrome [20]. The second patient was an 11-month-old boy from a consanguineous Tunisian family who presented with early-onset, recurrent episodes of fever, hyperpigmentation with hypertrichosis, dysmorphic features, hepatosplenomegaly and pericardial effusion [21]. In addition, Korbi et al. identified in a Tunisian patient, with a severe phenotype of H syndrome, a compound heterozygosity for the Arg363Gln and a splice site mutation c.300 + 1G > C in intron 2 of the *SLC29A3* gene [22]. Jaouadi et al. described a 23-year-old Tunisian man, with an Arg363Gln mutation in a homozygous state, presented with hyperpigmentation and hypertrichosis, inguinal and scrotal swelling and retracted penis [23]. As shown in our study, the Arg363Gln mutation seems to be the most frequent mutation among Tunisian patients with H syndrome. The medical history of our family highlights a wide phenotypic variability characterizing H syndrome, in terms of organ involvement and disease severity, and confirms that patients carrying the same mutation in the *SLC29A3* gene may display different symptoms.

It is well known that *SLC29A3* spectrum disorder is characterized by a poor phenotype–genotype correlation; thus, a given mutation may be responsible for different phenotypes [4, 5, 13]. For instance, the most common mutation, Gly437Arg mutation has been identified in patients with H syndrome, FHC, PHID or Familial RDD [3]. Although the phenotypes of Familial RDD and H syndrome are distinguished by a minimal clinical overlap, both may be associated with identical mutations of c.307delTT, c.1309G > A and c.1088G > A in *SLC29A3* [3, 8, 19].

In the current study, we identified two different phenotypes (Familial RDD/H syndrome) sharing the same c.1088G > A mutation in a single extended family. Our observation supports the idea of poor phenotype–genotype correlation in the *SLC29A3* mutations. As indicated in Table 3, it now seems that phenotypic heterogeneity is a common finding in *SLC29A3* disorders. In addition to our report, Spiegel et al. reported in an Israeli Moslem Arab family, a 28-year-old PHID woman, her 5-year-old nephew had features consistent with H syndrome, and her 23-year-old sister had a more severe phenotype combining features of both syndromes [6]. In this context, Al-Haggar et al. reported an 8-years Egyptian female who had overlapping features of H syndrome and PHID [13]. Jesus et al. reported a Moroccan girl born to consanguineous parents who presented with cumulative features of PHID, H syndrome, FHC and Familial RDD in whom a homozygous splice site mutation (c.300 + 1G > C) in the *SLC29A3* gene was identified [24].

**Table 3:** Phenotypic heterogeneity of *SLC29A3* gene mutations

SLC29A3 mutations	Origin	Intra-familial phenotypic variability	Intra-individual overlap syndrome	References
c.1279G > A, [p.Gly427Ser]	Israel	H syndrome/PHID:	–	Spiegel et al.
c.1309G > A, [p.Gly437Arg]				
c.1279G > A, [p.Gly427Ser]	Egypt	–	H syndrome/PHID	Al-Haggar et al.
c.300 + 1G > C	Morocco	–	H syndrome/PHID/MRD/FHC	Jesus et al.
c.1309G > A, [p.Gly437Arg]	Palestine	–	FHC/MRD	Rossbach et al.
				Morgan et al.
c.1088G > A, [p.Arg363Gln]	Tunisia	H syndrome/MRD	–	This report

PHID: Pigmented hypertrichosis with insulin-dependent diabetes mellitus syndrome; FHC: Faisalabad histiocytosis; RDD: Rosai–Dorfman disease

In 2 Pakistani brothers originally reported by Moynihan et al., Morgan et al. identified a homozygous splice site mutation (c.300 + 1G > A) of the *SLC29A3* gene [3, 25]. Additionally to the variable phenotypes between FHC and H syndrome, this mutation has also been found in patients diagnosed with H syndrome [8]. Furthermore, Wei et al. describe the crucial role of ENT3 as a mitochondrial and lysosomal nucleoside transporter in maintaining its integrity and suggest that ENT3 is an essential metabolite transporter that supports T cell homeostasis and activation. These findings support that some clinical variability between H syndrome and Familial RDD, such as splenomegaly and lymph nodes, is due to the lack of T cells homeostasis following the loss of lysosome integrity [27]. The present study emphasizes the previously reported intra-familial clinical heterogeneity among Tunisian patients with overlapping features of *SLC29A3* disorders (Familial RDD/H syndrome) even in patients who share the same *SLC29A3* mutation. This suggests the implication of other genetic and/or epigenetic regulatory factors that can influence the clinical expression of this observed variation [3–5].

## Conclusion

In conclusion, our study reveals that despite a single mutation, the clinical expression of the *SLC29A3* disorders may be significantly heterogeneous suggesting a poor genotype–phenotype correlation for the disease.

## Data Availability

Please contact the author for data requests.
